# Human organ chips for regenerative pharmacology

**DOI:** 10.1002/prp2.1159

**Published:** 2023-12-27

**Authors:** Girija Goyal, Chaitra Belgur, Donald E. Ingber

**Affiliations:** ^1^ Wyss Institute for Biologically Inspired Engineering at Harvard University Boston Massachusetts USA; ^2^ Vascular Biology Program and Department of Surgery Boston Children's Hospital and Harvard Medical School Boston Massachusetts USA; ^3^ Harvard John A. Paulson School of Engineering and Applied Sciences Harvard University Cambridge Massachusetts USA

**Keywords:** clinical predictivity, intestine‐on‐a‐chip, lung‐on‐a‐chip, lymph Node‐on‐a‐chip, microphysiological systems, organs on chips

## Abstract

Human organs‐on‐chips (organ chips) are small microfluidic devices that allow human cells to perform complex organ‐level functions in vitro by recreating multi‐cellular and multi‐tissue structures and applying in vivo‐like biomechanical cues. Human Organ Chips are being used for drug discovery and toxicology testing as an alternative to animal models which are ethically challenging and often do not predict clinical efficacy or toxicity. In this mini‐review, we summarize our presentation that reviewed the state of the art relating to these microfluidic culture devices designed to mimic specific human organ structures and functions, and the application of Organ Chips to regenerative pharmacology.

## Graphical abstract

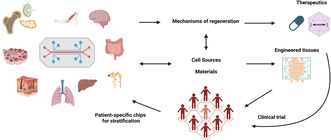

**Abbreviations**
AID: activation‐induced cytidine deaminase
ECM: extracellular matrix
EED: environmental enteric dysfunction
IND: investigational new drug
iPSCs: induced pluripotent stem cells
LF: lymphoid follicle
NHP: non‐human primate
Organ Chips: human organs on chips
RAGE: receptor for advanced glycation end products
TEER: trans‐epithelial electrical resistance

Our Organ Chip work began with the design of an optically clear and flexible two‐channel microfluidic chip where the channels are separated by a cell‐permeable membrane that can be stretched by applying a vacuum. This allowed us to mimic breathing motions in a Lung Alveolus Chip^1^ as well as the attachment and transmigration of circulating immune cells across an endothelial barrier in response to environmental pollutants or infection with respiratory pathogens. Using a similar design, we have now developed over 15 types of healthy and diseased human Organ Chips including models of lung small airway,^2^ intestine,^3^ liver,^4^ kidney,^5^, ^6^ skin, blood–brain barrier,^7^, ^8^ female genital tract,^9^, ^10^ bone marrow,^11^, ^12^ and lymph node,^13^ as well as non‐human primate (NHP), dog, rat,^14^ and mouse^15^, ^16^ Organ Chip models. We also have fabricated Organ Chips with a similar design using different materials (e.g., materials that exhibit less absorption of hydrophobic compounds) and we have integrated various types of sensors, including electrodes that measure trans‐epithelial electrical resistance (TEER) for real‐time quantification of tissue barrier function.^17^ We have used human Organ Chips to model various diseases, including rare genetic disorders,^11^ and to rapidly repurpose existing drugs for new applications (e.g., COVID‐19), as well as discover new therapeutics and carry out vaccine testing in vitro. As described in more detail below, these studies demonstrate that Organ Chip technology is sufficiently mature to meet the goals of regenerative pharmacology, which are to uncover the mechanisms of regeneration in different human tissues, elucidate the biological and engineering constraints for tissue‐engineered constructs, and conduct therapeutics testing (Figure 1). In addition to elucidating the pharmacodynamic properties of therapeutics, we have successfully replicated the pharmacokinetic profiles of AZD2811, a drug from Astra Zeneca, in the bone marrow chip^12^ and of nicotine and cisplatin in multi‐organ studies.^4^

**FIGURE 1:**
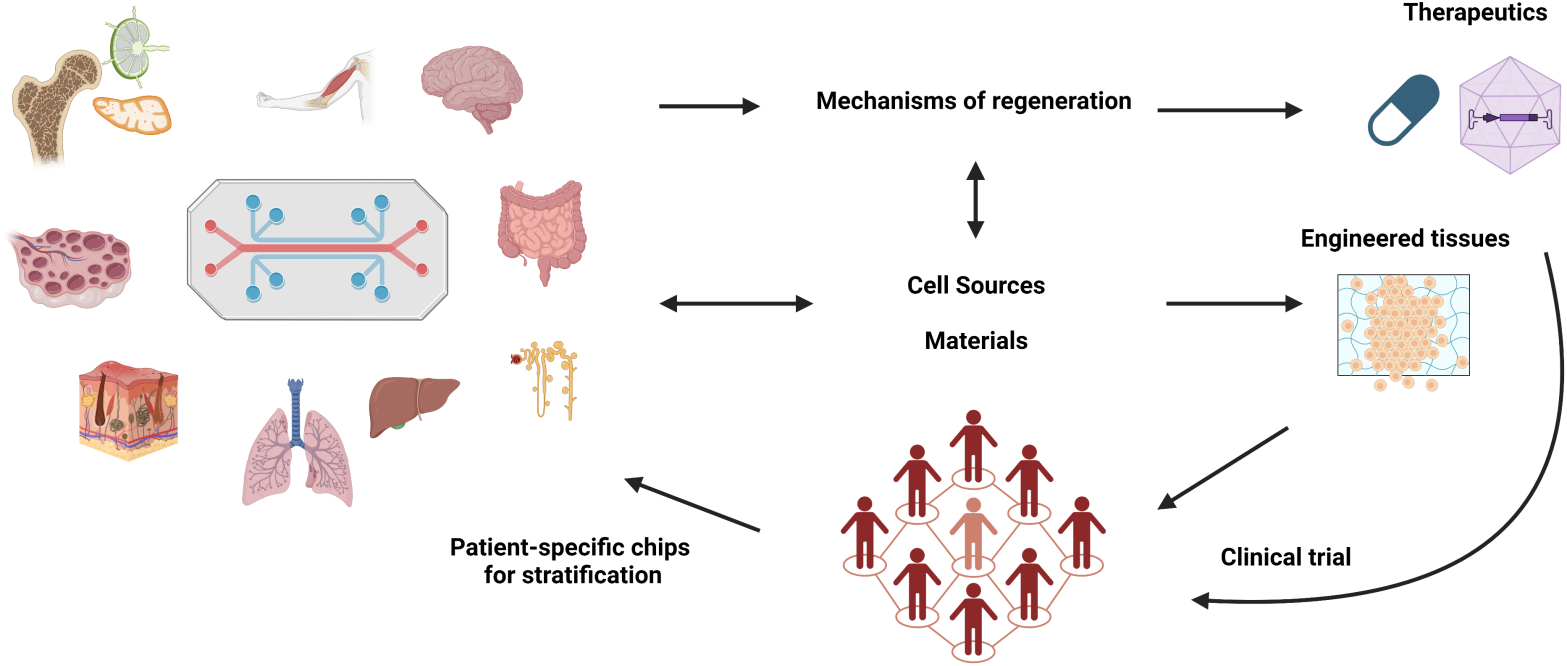
Human organs chips for regenerative pharmacology.

## 1 LUNG ALVEOLUS CHIP

The alveolus is a functional unit in the lung that contains a thin interface between inhaled air and the blood vessels that carry oxygen to the heart. This interface is made of epithelial cells facing the air and endothelial cells lining the blood vessel, which are separated by a specialized extracellular matrix (ECM) or basement membrane. Alveolar macrophages are loosely attached and in contact with the inhaled air; they have been extensively studied for their role in infections and other pathologies.^18^ Additionally, alveolar dendritic cells and fibroblasts have also been described.^19^ The alveolus expands and retracts with each breath and thus, it is critical to mimic not only the air‐liquid interface but the mechanical forces that human cells experience in this dynamic alveolar environment. We recently modeled influenza H3N2 infection in the Lung Alveolus Chip and found that physiological breathing motions reduce the infection and associated inflammation.^20^ Continuous exposure of Alveolus Chips to breathing motions resulted in increased levels of various interferon‐related antiviral genes and activation of host defense pathways while suppressing processes related to cell cycle and cell proliferation. Specifically, the S100A7 protein, which is a member of the S100 family and a ligand of the receptor for advanced glycation end products (RAGE), was produced by both alveolar epithelium and endothelium on‐chip.

As we found that S100A7 signals through RAGE, we explored whether RAGE inhibitors could suppress the host inflammatory response after viral infection on Alveolus Chips. Administration of the RAGE‐inhibiting drugs Azeliragon and FPS‐ZM1 inhibited the secretion of inflammatory cytokines when administered alone, and these drugs were synergized when each was administered in combination with the antiviral drug Molnupiravir.^20^ These data were used to support the submission of an investigational new drug (IND) application to the FDA to treat lung diseases.

Despite examples like these, skepticism remains about using Organ Chip technology alone for therapeutic validation, and thus studies in animal models such as NHP are commonly required. Although mandatory, these NHP models present many disadvantages for studying anti‐viral therapies due to their lack of appropriate virus‐induced pathology and to the wider variety of MHC genes in NHPs compared to those in humans. To overcome this barrier, we developed an NHP Lung Alveolus Chip using cells from Rhesus Macaque to compare human versus NHP tissue‐level responses to respiratory viral infection and therapeutics.

A pathology that could benefit from regenerative therapeutics is lung fibrosis observed after severe respiratory infections, idiopathic diseases, or radiation injury. Lung Alveolus Chips lined with alveolar epithelium and pulmonary endothelium showed extensive DNA damage, cellular hypertrophy, upregulation of inflammatory cytokines, and loss of barrier permeability 6 h after exposure to radiation. We identified that hemoxygenase‐1 (HMOX‐1) activity protects against acute injury but worsens radiation‐induced damage at later time points.^21^ We have also created a Cystic Fibrosis Lung Airway Chip model which can be used to identify therapeutics that facilitate regained lung function in Cystic Fibrosis patients.^2^

## 2 INTESTINE CHIP

Another organ that experiences repetitive movement throughout life is the human intestine. We have previously shown that primary human intestinal organoids if fragmented and seeded in our Organ Chip microfluidic devices form villi‐like structures and closely mimic the transcriptome of the human intestine.^22^ In recent years, implanted organoids have been shown to be capable of regenerating damaged intestine tissue in mouse models. The human Intestine Chip demonstrates that organoids can generate the absorptive enterocytes, goblet cells, chromogranin A‐producing enteroendocrine cells, and Paneth cells ex vivo even in the absence of stimuli from the microbiome, innervation, and other human cell types such as immune cells. We also engineered a human Colon Chip and, for the first time in vitro, showed that the model replicates the bi‐layer structure and thickness of the colonic mucus layer.^23^

The intestine is also the main microbiome reservoir in the human body. However, unlike the alveolus, the intestine nurtures many anaerobic microbes. To address this challenge, we built a companion chamber for the Intestine Chip which differentially aerates the medium flowing into the apical and basal channels allowing the chips to experience anaerobic conditions in the intestinal lumen while maintaining normoxic conditions in the vascular channel. Using this system, we demonstrated that it is possible to culture complex human microbiota composed of over 200 different bacterial species and strains, including anaerobic species, on the Intestine Chip.^23^ We have also modeled nutritional deficiency on‐chip with patient‐specific cells, which allowed us to model environmental enteric dysfunction (EED), a syndrome characterized by malnutrition and the establishment of an epigenetic state of malabsorption that prevents nutritional supplements from being effective. We showed that a deficiency in tryptophan and niacinamide leads to villus blunting, which severely reduces the absorptive area, the number of specific absorptive cells, and the mucus barrier, as is observed in EED patients. This was accompanied by an increase in the secretion of several inflammatory molecules and an enhanced leakiness of the gut barrier. Human Intestine chips created with cells from EED patients that are exposed to nutritional deficiencies also more closely mimic the transcriptomic signature of human disease than was previously possible.^3^

Studies to identify therapeutics that facilitate intestinal regeneration in EED Intestine Chips are ongoing. Regeneration of the intestine is also important for many other diseases including celiac disease and short bowel syndrome. Current approaches to correct intestinal dysfunction rely on therapeutics for reducing inflammation; managing the microbiota through diet, probiotics, antibiotics, or fecal matter transfer; and in extreme cases, performing an intestinal transplant. Most of these approaches are only partially successful and some carry extreme risk. The discovery and implementation of safe and effective therapeutics to stimulate the regeneration of intestinal tissue and restore function would bring great advances to the field of gastrointestinal health.

## 3 LYMPH NODE CHIP

By mimicking the tissue‐like density, ECM, and dynamic fluid flow that are experienced by lymph nodes in vivo, we were able to create a Lymph Node Chip that can reprogram circulating blood‐derived human lymphocytes to self‐organize into lymphoid follicles (LFs) characterized by the expression of activation‐induced cytidine deaminase (AID), which is normally absent in circulating lymphocytes.^13^ We showed that these chips mimic many aspects of the recall response to influenza such as the induction of IgG, formation of plasma cells, and the presence of cytokine biomarkers found in human serum. In unpublished work, we have further optimized the human Lymph Node Chip model to mimic IgG production in response to primary immunization with a previously unseen antigen. From a regenerative pharmacology perspective, an interesting finding was the requirement of dynamic fluid flow for the formation of LFs, which is reminiscent of the coevolution of blood vessels and the lymph node anlagen.^24^, ^25^

Most microphysiological models of the lymph node have focused on infectious disease or cancer vaccines; however, lymph node tissue mass and function are reduced in many diseases and/or with advancing age. In cancer patients, lymph nodes that contain cancer metastases are often removed. In patients with primary immunodeficiencies, lymph nodes can be defective or absent. Our future goal is to leverage the human Lymph Node Chip to develop therapeutics that enable patients to retain and improve lymph node function to prevent aging and disease‐related decline in immunity.

## 4 CHALLENGES AND ALTERNATIVES

Organ Chips have become increasingly complex, relying on patient‐derived primary cells, organoids, or induced pluripotent stem cells (iPSCs), which have allowed them to become increasingly predictive of human responses.^26^ However, the acquisition of high‐quality primary human cells and validation of functionality in vitro remains a challenge; and while iPSCs can form multiple cell types, they lack disease‐relevant epigenetic signatures and rarely achieve the functional fidelity of naturally and fully differentiated adult tissues. The COVID‐19 pandemic also revealed a lack of alternatives and resilience in the research supply chain. For instance, Matrigel™, a cell‐derived ECM used by many researchers, was in short supply during the pandemic. An alternative approach to Organ Chip modeling is the use of “slice culture” where slices of human organs are cultured on scaffolds to study organ‐level function ex vivo. However, this limits the number of users to those who have access to the tissues, and often tissues from patients are not available. Organ Chips may have limited utility for direct transplantation into humans, but they have the potential to inform tissue engineering studies. Conversely, the small tissue mass required by Organ Chips enables laboratory experimentation at a scale similar to routine tissue culture. Finally, the mechanistic understanding of a tissue in an Organ Chip may be skewed by the cell types represented on the chip. It is important to define the hypotheses and understand the limits of the model system when designing Organ Chip experiments and interpreting the results. However, to our knowledge, there are no other in vitro models that enable long‐term co‐culture of a microbiome with living human cells in an organ‐relevant context or that permit users to faithfully replicate clinically relevant drug exposure profiles (pharmacokinetics) as Organ Chips do.

## 5 LOOKING AHEAD

The examples discussed here show that Organ Chips can reveal the mechanisms of regeneration in different human tissues allowing us to design and test new therapeutics targeting regeneration of human tissues. Further, as they represent miniaturized organs or tissue‐tissue interfaces, they can help to define design criteria or materials for engineered organs such as biopolymers or cellular compositions. Regeneration of tissues goes beyond classical applications such as organ replacements; it is central to healing from any disease. It is difficult for clinical trials or even animal studies to provide data on how well the tissue is regenerating with a high resolution; for instance, researchers can only request a limited number of biopsies. With Organ Chips, for the first time, we can mimic healing and regeneration and observe it closely using the latest advances in microscopy and multiomics.

## 6 NOMENCLATURE OF TARGETS AND LIGANDS

Key protein targets and ligands in this article are hyperlinked to corresponding entries in http://www.guidetopharmacology.org, the common portal for data from the IUPHAR/BPS Guide to PHARMACOLOGY (Harding et al., 2018), and are permanently archived in the Concise Guide to PHARMACOLOGY 2019/20 (Alexander et al., 2019).

## AUTHOR CONTRIBUTIONS

Girija Goyal, Chaitra Belgur, and Donald E. Ingber reviewed past literature and wrote this manuscript.

## CONFLICT OF INTEREST STATEMENT

DEI. is a founder, board member, scientific advisory board chair, and equity holder in Emulate, Inc.; CB is a former employee of Emulate, Inc. and holds equity interests in Emulate Inc. GG, CB, and DEI are inventors on relevant patent applications.
